# A challenging STEC strain isolation from patients’ stools: an O166:H15 STEC strain with the *stx2* gene

**DOI:** 10.1128/spectrum.00098-24

**Published:** 2024-05-30

**Authors:** Surangi H. Thilakarathna, Vincent Li, Linda Chui

**Affiliations:** 1 Department of Laboratory Medicine and Pathology, Faculty of Medicine and Dentistry, University of Alberta, Edmonton, Canada; 2 Alberta Precision Laboratories - Public Health Laboratory (ProvLab), Edmonton, Canada; Michigan State University, East Lansing, Michigan, USA

**Keywords:** Shiga toxin-producing *Escherichia coli *(STEC), immunomagnetic separation, culture, *stx2*, non-O157:H7, O166:H15

## Abstract

Two patients with acute gastroenteritis tested positive for Shiga toxin-producing *Escherichia coli* (STEC) by polymerase chain reaction (PCR), and both strains carried the Shiga toxin 2 encoding gene. Since routine culture using CHROMagar STEC failed to recover these isolates, immunomagnetic separation (IMS) targeting the top six non-O157:H7 serotypes was used for isolate recovery. After two subsequent IMS runs, the STEC strains were isolated from trypticase soy broth with and without overnight enrichment for runs 1 and 2, respectively. Serotyping based on whole-genome sequencing revealed that both patients carried the strain O166:H15 STEC with the *stx2* gene. Hence, the magnetic beads used in IMS appeared to have cross-reactivity with other *E. coli* serotypes. When the STEC isolates from both stools were cultured on CHROMagar STEC and sheep blood agar (BAP), two distinct colony sizes were apparent after overnight incubation. The small and large colonies were picked and separately cultured on both media, and colony growth was observed for 2 weeks at room temperature after an initial overnight incubation at 37°C. After 1 week, the colonies showed concentric ring structures with a darker center and a lighter surrounding on CHROMagar STEC and a “fried egg”-resembling structure with a raised circular center and a flat surrounding on BAP. Both colony types remained morphologically different on CHROMagar STEC throughout the 15 days. However, on BAP, their appearance was comparable by day 7.

**IMPORTANCE:**

Shiga toxin-producing *E. coli* (STEC) infections can lead to severe complications such as bloody diarrhea and hemolytic uremic syndrome (HUS), especially in young children and the elderly. Strains that carry the shiga toxin 2 gene (*stx2*), such as O157:H7, have been mostly linked with severe disease outcomes. In recent years, outbreaks caused by non-O157:H7 strains have increased. *E. coli* O166:H15 has been previously reported causing a gastroenteritis outbreak in 1996 as a non-STEC strain, however the O166:H15 serotype we recovered carried the *stx2* gene. It was particularly challenging to isolate this strain from stools by culture. Consequently, we tested immunomagnetic separation for the STEC recovery, which was a novel approach on clinical stools. Virulence genes were included for the characterization of these isolates.

## OBSERVATION

Shiga toxin-producing *Escherichia coli* (STEC) is a group of pathogenic Gram-negative bacteria that carries the bacteriophage-encoded virulence genes, *stx1* and/or *stx2,* to produce potent Shiga toxin 1 and Shiga toxin 2, respectively (1). Different STEC strains can carry either one *stx* gene or both (1). STEC that carry *stx2,* such as O157:H7, are considered “high-risk” as they are linked with complications like bloody diarrhea and hemolytic uremic syndrome (HUS) (2–4). In recent years, highly pathogenic non-O157:H7 STEC strains have emerged and caused outbreaks (5, 6). Some of these infections have led to severe disease outcomes (6, 7). Therefore, it is crucial to study the new emerging STEC strains, especially when they carry *stx2* (2).

Serotyping and cluster analysis are crucial for public health surveillance and gastroenteric outbreak detection. The recovery of STEC isolates from patients’ stools is essential for these purposes as well as for further characterizations. Recovering the isolates through culture is labor-intensive and mostly challenging as not all non-O157 STEC would grow on selective media when directly inoculated from stools (8–10). Therefore, it is crucial to investigate alternate approaches to recover these pathogens from patients’ stools. This is a short report on the successful isolation and characterization of an uncommon serotype of O166:H15 STEC from two cases from Alberta, Canada, which were particularly challenging to isolate through culture alone.

Two patients’ stool specimens: P-1 and P-2 from Alberta, Canada, submitted due to acute gastroenteritis, tested positive only for STEC by polymerase chain reaction (PCR) using the BD Max enteric bacterial panel targeting STEC, *Salmonella* spp., *Shigella* spp./ enteroinvasive *E. coli* (EIEC), and *Campylobacter* spp. These specimens were sent to Alberta Precision Laboratory-Provincial Laboratory for Public Health (ProvLab) for pathogen isolation and identification. ProvLab reported both stools were PCR-positive for *stx2* but failed to isolate the strains through routine culture. Consequently, these samples were referred to ProvLab Chui research laboratory for further investigations. Upon receipt, 10 µL of stools was enriched in trypticase soy broth (TSB) overnight at 37°C. Stools and the enriched TSB cultures were inoculated onto CHROMagar STEC and MacConkey agar (MAC) plates (Dalynn Biologicals, Calgary, AB, Canada) and were incubated overnight at 37°C. Both samples showed growth only on MAC plates, and colony sweeps tested PCR-positive for *stx2*. The colonies were small and light pink in color and were embedded within off-white-colored commensal bacterial colonies (PCR-negative for *stx*). The nature of the colony growth and pinpoint size complicated STEC isolation through culture. Multiple attempts were made to recover pure colonies through TSB, MAC, and onto CHROMagar STEC culture, but failed.

**Fig 1:**
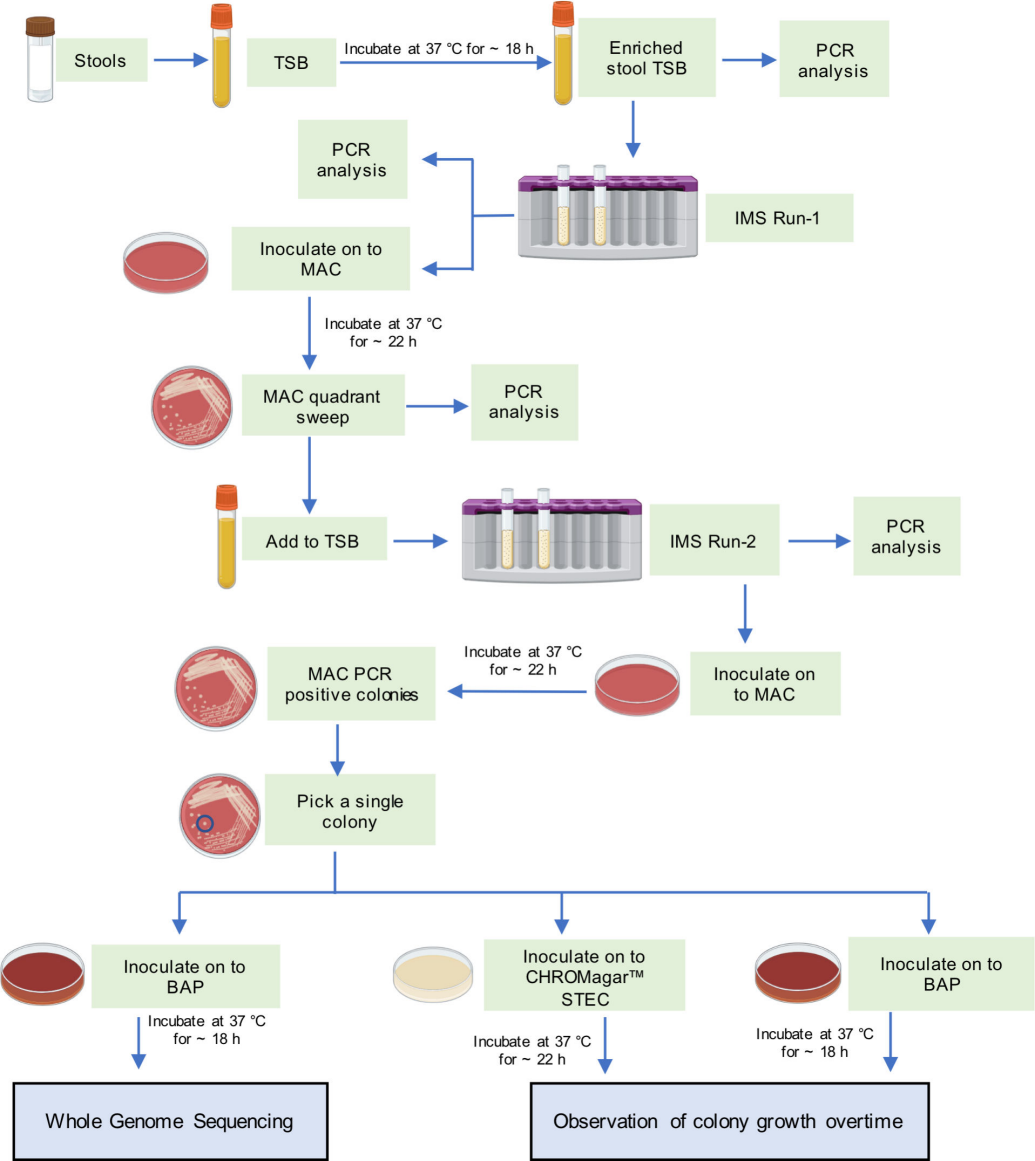
Steps followed for STEC O166:H15 isolation. TSB: trypticase soy broth; PCR: polymerase chain reaction; MAC: MacConkey agar; IMS: immunomagnetic separation; BAP: sheep blood agar; STEC: Shiga toxin-producing *E. coli*.

Considering these STEC strains carrying *stx2* showed no growth on CHROMagar STEC and proved challenging to isolate, there was a possibility that these were not O157:H7 strains. Consequently, they were presumed to be in the top six non-O157:H7 category. As isolation through culture was difficult, the immunomagnetic separation (IMS) method was explored as an alternative approach for the recovery. In IMS, a pool of immunomagnetic beads (RapidCheck CONFIRM STEC IMS, Romer Lab Division Holdings GmbH, Getzersdorf, Austria) that target O26, O45, O103, O111, O121, and O145 STEC are used to separate these STEC from the other background bacteria in a sample. A common application of IMS is to isolate the top six STEC in contaminated food products (11), but its usage to recover STEC from stools has not been previously documented. The flow chart (Fig. 1) illustrates the STEC isolation process that was followed.

IMS was applied on enriched stool TSB according to the manufacturer’s instructions with slight modifications. Pea-sized amounts of stools from P-1 and P-2 were separately inoculated in 3.5 mL TSB and were incubated at 37°C overnight. A 1-mL aliquot from each of these enriched TSB were treated with 51 µL of pooled immunomagnetic beads, washed three times with Tween–PBS, and then resuspended in 100 µL of Tween–PBS (a protocol used for immunocapture in routine food laboratory in ProvLab). A 50-µL aliquot was subjected to lysis to obtain the DNA for PCR analysis (12). The remaining 50-µL aliquot was inoculated onto MAC plates. STEC-captured beads from P-1 and P-2 tested PCR-positive for *stx2*. The colony morphologies from both samples were similar on MAC plates, and sweeps from all four quadrants tested positive for *stx2*. However, a mixed culture of small pink and large off-white colonies was observed, indicating the cross-reactivity of the top six non-O157 immunocapture beads. The small pink colonies tested positive for *stx2* but not the larger ones. Consequently, the recovery of pure STEC colonies was not possible at this stage. Therefore, a second IMS was carried out using a sweep of colonies from the first IMS-MAC agar. P-1 and P-2 MAC colony sweeps were added separately to 1.5 mL of TSB, and 1 mL of the mixture was retrieved for the IMS protocol as described previously. After the separation, the beads were spread on MAC plates and incubated overnight (~20 h). On the following day, although large off-white colonies were still present, numerous light pink isolated colonies were also found and were confirmed *stx2-*positive by PCR.

Single *stx2-*positive colonies from P-1 and P-2 MAC agar were picked and inoculated onto sheep blood agar plates (BAP) and were incubated overnight at 37°C. The colonies from P-1 were larger, while the colonies from P-2 exhibited two different sizes on BAP. To determine the serotypes, the large colonies from P-1 and small and large colonies from P-2 were separately inoculated onto new BAP. After overnight incubation, DNA was extracted from colony sweeps and WGS was performed according to the PulseNet Canada protocol (13), and serotypes were determined by the ECTyper (14). Sequence analysis and genome assembly were performed using the bioinformatics pipeline pathogenseq (https://github.com/xiaoli-dong/pathogenseq). Briefly, pathogenseq assesses sequence quality using fastqc v0.11.9 (https://github.com/s-andrews/FastQC) and seqkit v2.6.0 (15). Host sequences were removed from the raw WGS data using Hostile (https://github.com/bede/hostile). Adapter and quality trimming were performed using fastp v0.23.4 (16). De-hosted and trimmed sequences were assembled using Shovill v1.1.0 (https://github.com/tseemann/shovill) and the SPAdes v3.14.0 assembler under default settings. Genome assembly quality was assessed using CheckM2 v1.0.1 (https://github.com/chklovski/CheckM2). All three isolates from P-1 (NCBI accession no: SAMN40994153) and P-2 (NCBI accession no: SAMN40994154 [small colonies] and SAMN40994155 [large colonies]) were identified as STEC O166:H15. Table 1 shows the quality statistics of WGS analysis.

**TABLE 1:** Quality statistics of WGS analysis

Accession no.	Mapped reads	Mapped bases	Contig count	Contig N50	Genome size	Coverage depth
SAMN40994153	1,641,937	41,7335,872	89	258,415	5,158,670	81
SAMN40994154	1,896,192	509,793,253	71	295,058	5,110,785	100
SAMN40994155	2,537,943	648,122,428	65	295,058	5,117,644	127

Virulence factor detection was performed on the resulting genome assemblies using AbritAMR v1.0.15 (https://github.com/MDU-PHL/abritamr) and AMRFinder v3.12.8 (https://github.com/ncbi/amr; AMRFinderPlus database version 2024–01-31.1) (17) with default settings and the species set as “*Escherichia.*” The identified virulence factors are shown in Table 2 and were observed in all three isolates (17).

**TABLE 2:** The virulence factors identified in the O166:H15 strains^*a*^

Gene symbol	Name of gene
*eilA*	HilA family transcriptional regulator EilA
*espX1*	Type III secretion system effector EspX1
*fdeC*	Inverse autotransporter adhesin FdeC
*iucA*	Aerobactin synthase IucA
*iucB*	N(6)-hydroxylysine O-acetyltransferase IucB
*iucC*	NIS family aerobactin synthetase IucC
*iucD*	NADPH-dependent L-lysine N(6)-monooxygenase IucD
*iutA*	Ferric aerobactin receptor IutA
*papA*	P fimbrial usher protein PapA
*papC*	P fimbrial minor subunit PapC
*papH*	P fimbrial major subunit PapH
*sslE*	Lipoprotein metalloprotease SslE
*stxA2o*	Shiga toxin Stx2o subunit A
*stxB2o*	Shiga toxin Stx2o subunit B

^*a*^ The listed virulence factors were present in both small and large colonies.

**Fig 2:**
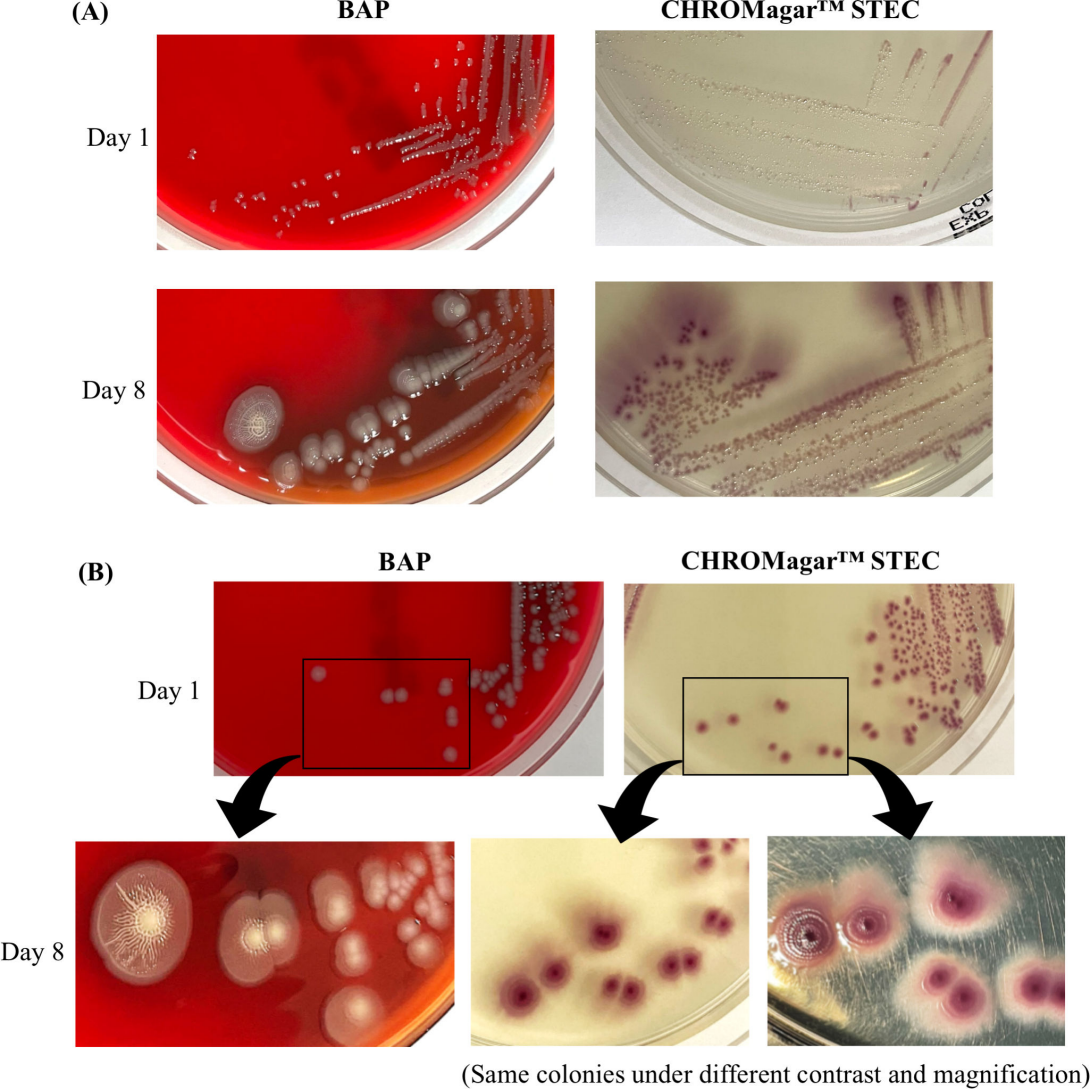
Colony morphology of O166:H15. The morphology of the (**A**) small and (**B**) large O166:H15 colonies on sheep blood agar and CHROMagar STEC at day 1 and day 8. The same large colonies on CHROMagar STEC are displayed under different contrasts to emphasize on the concentric ring structures.

To observe the colony growth over time, colonies from P-1 and P-2 were inoculated onto CHROMagar STEC and BAP. Plates were incubated at 37°C overnight and then left at room temperature until day 15. The small P-2 colonies grew fine colorless colonies that were barely visible on day 1 on both types of media (Fig. 2A). These colonies became visible after day 4 at room temperature. The large colonies from P-1 and P-2 showed similar morphologies on each type of media on day 1. By day 8, both large and small colonies on BAP resembled “fried egg”-like structures with a raised circular center and a flat surrounding (Fig. 2A and B), and ridges were radiating from the circular center. On CHROMagar STEC, the small colonies remained smaller with a pinpoint center (Fig. 2A) and the large colonies further expanded in size and exhibited concentric ring structures with a darker center (Fig. 2B). The colonies on both BAP and CHROMagar STEC developed diffused outer layers during days 8 to 15, and the colony morphology did not significantly change on each media over this period. In parallel, a STEC O157:H7 was inoculated onto CHROMagar, MAC, and BAP, and the colony morphologies were not comparable to those of O166:H15 under the same conditions and time duration (data not shown).

In this investigation, we aimed to report this uncommon case of *E. coli* O166:H15 STEC and to emphasize the challenges faced in the recovery of these isolates from stools. We tested IMS as an alternative method to using culture alone, which was a novel approach to recover STEC from clinical stools. Although the immunomagnetic beads targeted the top six serotypes, the cross-reactivity allowed us to recover this O166:H15 serotype, which is not from the top six serotypes. A gastroenteritis outbreak pertaining to *E. coli* O166:H15 was previously reported in Osaka, Japan, in 1996 (18). However, this particular O166:H15 strain was not considered an STEC as it did not carry any *stx* genes. Since the O166:H15 STEC we isolated carried *stx2,* it has a great potential to cause complications such as HUS in patients. Unfortunately, the patient chart reviews were not available, so disease outcomes could not be discussed here.

## Data Availability

The genome sequences of STEC isolates has been deposited to the NCBI repository. The accession numbers of the STEC isolates are as follows: P1 colonies, SAMN40994153; P2 small colonies, SAMN40994154; and P2 large colonies, SAMN40994155.
